# Sarco/endoplasmatic reticulum calcium ATPase activity in healthy muscle and Brody disease

**DOI:** 10.1016/j.bbrep.2026.102554

**Published:** 2026-03-24

**Authors:** J.P. Molenaar, M.M. Snoeck, S. Treves, B.J. Stoltenborg, K.C. Wethly, A. Oosterhof, E.J. Kamsteeg, D. Sternberg, J. Doorduin, B.G. van Engelen, N.C. Voermans, R.J. Rodenburg

**Affiliations:** a Department of Neurology, Donders Institute for Brain, Cognition and Behaviour, Radboud University Medical Center, Nijmegen, the Netherlands; b Department of Neurology, Rijnstate, Arnhem, the Netherlands; c Department of Anesthesiology, Canisius-Wilhelmina Hospital, Nijmegen, the Netherlands; d Department of Biomedicine, University Hospital Basel, Basel, Switzerland; e Department of Life Sciences, University of Ferrara, Ferrara, Italy; f Translational Metabolic Laboratory, Department of Pediatrics, Radboud Centre for Mitochondrial Disorders, Nijmegen, the Netherlands; g Department of Biochemistry, Radboud Institute for Molecular Life Sciences, Radboud University Medical Center, Nijmegen, the Netherlands; h Department of Human Genetics, Radboud University Medical Center, Nijmegen, the Netherlands; i Service de Biochimie Métabolique, Centre de génétique moléculaire et chromosomique, Hôpitaux Universitaires Pitié-Salpêtrière, Paris, France

**Keywords:** Sarco-/endoplasmatic reticulum calcium ATPase, Brody disease, ATP2A1 gene

## Abstract

The sarco-/endoplasmatic reticulum calcium ATPase (SERCA) is a calcium transporter that plays a key role in the excitation-contraction-relaxation cycle by enabling muscle relaxation. Pathogenic variants in the SERCA-encoding *ATP2A1* gene cause Brody disease, a rare recessive myopathy with exercise-induced muscle stiffness as the main feature. Measurement of SERCA activity is an important in vitro functional assay to aid in confirming the pathogenicity of variants in this gene. Furthermore, measurement of SERCA activity is used to study physiological and pathological excitation-contraction (de)coupling. For both, a robust assay is of the utmost importance. We aimed to fully reassess the SERCA activity assay, establish new reference values, and validate the assay by measurement of Brody disease muscle samples.

All different SERCA assay parameters were extensively re-evaluated and optimized, including time linearity, the effect of sonication and pH, and the effects of homogenate, KCl/Imidazole, ATP, MgCl_2_, and CaCl_2_ concentrations. With the optimized assay, SERCA activity was assessed in muscle samples from healthy controls (*n* = 28) and patients with Brody disease (*n* = 4).

We were able to provide new reference values and demonstrate marked decreased SERCA activity in Brody disease muscle samples (30.0 ± 4.2 mU/mg protein) compared to controls (86.7 ± 25.1 mU/mg protein). We developed a robust enzyme assay to measure SERCA activity with high discriminative power to distinguish patients with Brody disease from controls. Thus, this assay provides a reliable method of studying this important calcium pump for both clinical and scientific purposes.

## Highlights

• We developed a robust assay to measure SERCA activity in human skeletal muscle.
• Assay was validated by detecting decreased SERCA activity in Brody disease samples.
• Assay can help confirm pathogenicity of *ATP2A1* variants for Brody disease diagnosis.

## Graphical abstract

**Figure:**
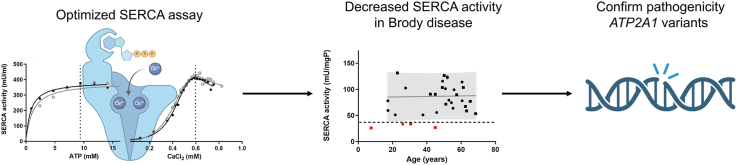

## 1 Introduction

The sarco-/endoplasmatic reticulum calcium ATPase (SERCA) is an important calcium transporter located in the membrane of the sarcoplasmatic reticulum (SR) [1]. It plays a key role in the excitation-contraction-relaxation cycle by enabling muscle relaxation. When an action potential travels along the membrane of the sarcolemma, it will activate the dihidropyridine receptor located in the T-tubules. This in turn activates the ryanodine receptor type 1 (RyR1) Ca^2+^ channel, leading to the efflux of calcium ions from the SR into the myoplasm. Calcium can then bind to the actin molecules, initiating the cross-bridge cycle and allowing for muscle contraction. Finally, muscle relaxation is achieved mainly by the reuptake of calcium into the SR by ATP dependent SERCA [2,3].

The two main isoforms of SERCA in human skeletal muscle cells are SERCA1 mainly expressed in fast twitch (type II) muscle fibers, encoded by the *ATP2A1* gene, and SERCA2 mainly expressed in the slow twitch (type I) muscle fibers, encoded by the *ATP2A2* gene [1]. Pathogenic homozygous or compound heterozygous variants in *ATP2A1* cause Brody disease, a rare myopathy mainly characterized by exercise-induced muscle stiffness [4]. Dysfunction of SERCA2 caused by *ATP2A2* gene mutations, leads to a rare dominant skin condition known as Darier disease, but has not been associated with a myopathy thus far [[5], [6], [7]].

When *ATP2A1* genetic variants of unknown clinical significance are identified in suspected Brody disease patients, SERCA activity measurements can provide supporting evidence regarding their pathogenicity [4]. Furthermore, measurements of SERCA activity are used to study physiological muscle function and calcium handling in myopathies, for instance, to study compensatory mechanisms of calcium homeostasis. For both purposes, it is important to have a robust assay with high discriminative power available.

In 1992, a method to measure SERCA activity was developed in our center [8,9]. In that assay, compound SERCA (SERCA1 + SERCA2) activity was measured in whole muscle homogenates obtained from quadriceps muscle samples using a molybdate-Fe(II) Pi assay [9]. SERCA1 is the predominant isoform in quadriceps muscle from individuals with no underlying myopathy, making compound SERCA activity a reliable estimate of SERCA1 activity [8,10]. The activity of SERCA is expressed as the calcium dependent conversion of ATP to ADP normalized to the amount of total protein (mU phosphate/mg protein). We used the molybdate-Fe(II) Pi assay over methods like the NADH-coupled enzyme assay (e.g., Pyruvate Kinase/Lactate Dehydrogenase) due to its better suitability in a clinical diagnostic setting, where simplicity, ease of implementation, and high-throughput capability are essential. The method is highly feasible for diagnostic lab implementation and can typically be completed within approximately 4 h, depending on sample load.

In other studies, either largely similar approaches, e.g., Refs. [[11], [12], [13], [14], [15], [16]], or alternative techniques, e.g., Refs. [[17], [18], [19], [20], [21]], have been used to assess SERCA activity in skeletal muscle. The latter include the measurement of calcium dependent Pi release in HEK-293 cells transfected with SERCA [17,19], separating the SR and isolating the SERCA protein from the rest of the muscle homogenate [20], or measurement of calcium uptake in individual skinned muscle fibers [18]. However, these alternative techniques tend to be more time consuming and require specialized equipment and techniques not commonly available in a clinical diagnostic biochemical laboratory.

The goal of this study is to set up a robust method that can identify decreased SERCA activity. For this, we first fully reassessed the previously published enzyme assay, including time linearity, the effect of sonication and pH, and the effects of homogenate, KCl/Imidazole, ATP, MgCl_2_, and CaCl_2_ concentrations. Second, with this optimized assay we established new reference values using muscle samples from healthy subjects; and third, we validated the assay by measuring muscle samples from genetically confirmed Brody disease patients.

## 2 Materials and methods

### 2.1 Sample collection and handling

Separate ethics committee approval was not required, as the biopsy samples were obtained as part of routine diagnostic procedures. Patients had provided written and/or verbal consent for the use of residual material not required for diagnostic purposes for research, in accordance with Dutch regulations on the use of leftover human tissue. The muscle samples from healthy controls were mostly acquired from subjects that previously underwent quadriceps muscle biopsy to exclude malignant hyperthermia (MH) susceptibility (*n* = 23) because of a family member with a (suspected) MH event. They had normal results on the in vitro contracture test, normal CK levels, no neuromuscular symptoms (myalgia, cramps, etc), no thyroid disease, and no *RYR1* mutations (the main gene involved in MH susceptibility) [22]. Additionally, we included quadriceps samples from healthy subjects that were originally acquired for a different study (*n* = 5) and for which separate ethical approval was obtained from the Medical Ethics Review Committee region Arnhem-Nijmegen (approval number 2011/181) [23]. The Brody disease samples (*n* = 4) were biopsied in a diagnostic setting, acquired either from quadriceps muscle (*n* = 2) or deltoid muscle (*n* = 2). All samples were snap frozen immediately after collection and stored at −80 °C.

### 2.2 Analytical methods

For SERCA measurements, 20 mg of frozen muscle tissue was homogenized in 1 ml ice-cold homogenization buffer (250 mM sucrose, 2 mM ethylenediaminetetraacetic acid (EDTA), 10 mM Tris Cl, pH 7.4) using a potter-Elvehjem homogenizer, after which aliquots of the muscle homogenate were stored at −80 °C [11,24]. Before the measurements, the samples were freeze-thawed three times followed by sonication for 10 s at 20% amplitude. Samples were kept on ice during sonication to minimize heat generation. Protease inhibitors or reducing agents were not included in our homogenization protocol, as we found no significant improvement in SERCA stability with their use. The SERCA reaction mix consisted of 0.47 mM ethylene glycol tetraacetic acid (EGTA), 4.7 mM MgCl_2_, 9.4 mM adenosine triphosphate (ATP), 0.588 mM CaCl_2_, 94.1 mM KCl, 94.1 mM imidazole, pH 7.4. For background measurements, the same mixture without CaCl_2_ was prepared. To 400 μl of this mixture, 25 μl of 20 mg/mL muscle homogenate was added, and incubated at 37 °C for 120 min, after which the reaction was stopped by adding 1.5 ml of ice cold 8.6% trichloroacetic acid. The above mentioned assay represents the optimized conditions which were determined by varying different parameters, as described in the results section. These parameters were used unless specified otherwise. The goal was to optimize reaction conditions to detect differences in SERCA activity. As such, the use of only two samples was intended to establish preliminary parameters to guide further optimization.

The free phosphate formed during the enzyme reaction was measured using spectrophometry (Thermo Fisher Konelab 20XTi autoanalyzer) after adding a coloring reagent, consisting of 0.66 M H_2_SO_4_ + 1.15% (w/v) ammonium heptamolybdate, to which 0.33 M of Fe(II)SO_4_ was added prior to use. The phosphate concentrations were calculated using a calibration curve prepared with a KH_2_PO_4_ concentration series. Protein content (mgP) in the homogenate was spectrophotometrically assessed by the U/CSF protein test for Konelab (Thermo Scientific, article 981843) following the manufacturer's instructions.

To determine within-sample variability of sonicated and not-sonicated samples, SERCA activity of 22 samples from healthy subjects were measured in duplicate with and without additional sonication of the homogenate. To assess interday variability, two samples were processed (all the previously mentioned steps, starting from the frozen whole muscle homogenate) and measured on seven (sample 1) or 10 (sample 2) separate days. To determine intraday variability, two samples were measured three times at 2 h intervals.

### 2.3 Data analysis

Mean values were calculated of all duplicate measurements and further analyzed with Excel (Microsoft Office 2007, USA) and Prism (GraphPad Software, version 5.03, USA). Within-sample variability was expressed as the overall within-sample coefficient of variation (CV), which was calculated as the mean ± SD of all individual CVs (standard deviation divided by the mean of the duplicate measurements). Inter- and intraday variability were also expressed as CVs. Two-tailed paired t-tests were used to compare variability of sonicated versus non-sonicated measurements. The Shapiro-Wilk test was used to check if SERCA activity in controls was normally distributed. To test for differences between sexes, unpaired t-tests were used. For all tests, a *P*-value <0.05 was considered significant. Data are described as means ± SD.

## 3 Results

In this study, muscle samples from 32 subjects were examined. The characteristics of the subjects are given in Table 1. The male and female control subjects had a similar age (*P* = 0.117).

**Table 1:** **|** Subject characteristics and SERCA activity.

	Sex	Age at biopsy	SERCA activity (mU/mgP)	Genotype	*ATP2A1* variants	Mutation type	Classification pathogenicity
**Healthy controls**	M (*n* = 12)	53.2 ± 12.8	88.6 ± 24.7				
	F (*n* = 16)	44.4 ± 15.2	85.6 ± 25.7				
	All (*n* = 28)		86.7 ± 25.1				
**Brody disease**	M (*n* = 3)	7.5	26.1	Homozygous	c.100G > T p.Glu34∗	Stop	5
		30.6	33.8	Homozygous	c.490C > T p.Arg164∗	Nonsense	5
		44.9	26.9	Homozygous	c.2366C > T p.Pro789Leu	Missense	5
	F (*n* = 1)	26.0	33.5	Compound heterozygous	c.(928 + 1_929-1)_(1094 + 1_1095-1)del c.1811G > A p.Arg604His	Large deletion Missense	5 4
	All (*n* = 4)	27.2 ± 15.4	30.0 ± 4.2				

Mutations are numbered according to transcript NM_004320.4.

The international classification of pathogenicity was used which ranges from 1 (benign variant), through 4 (likely pathogenic), to 5 (pathogenic) [25].

mgP = milligrams of protein; SERCA = sarcoplasmic/endoplasmic reticulum ATPase.

### 3.1 SERCA assay optimization

**Fig. 1:**
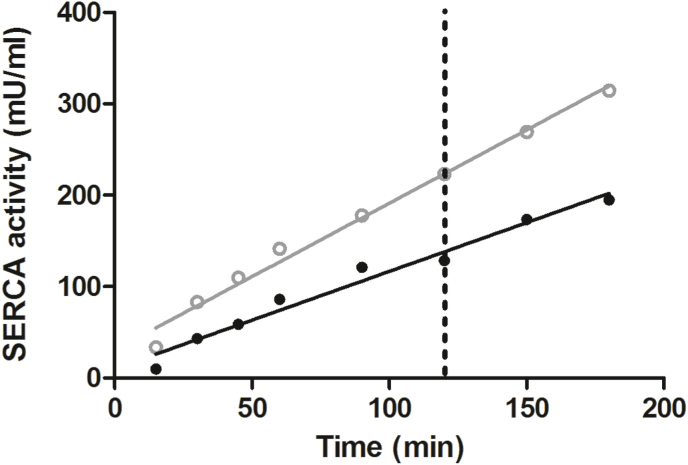
Effect of incubation time on SERCA activity. Two muscle samples were measured, both showing significant slope deviation from zero with P = <0.0001 and r^2^ = 0.973 for sample 1 (black circles and line) and P = <0.0001 and r^2^ = 0.988 for sample 2 (grey circles and line). The dotted line represents the chosen incubation time for future measurements (120 min).

There was a linear relationship between SERCA activity and incubation time (Fig. 1). On the basis of these results, an incubation time of 2 h was chosen for subsequent measurements because this results in well detectable activities and falls within the linear range of the assay.

The mean CV was lower in the sonicated homogenates compared to the non-sonicated homogenates (7.4 ± 6.8 vs 24.3 ± 22.3%, respectively; *P* = 0.001), indicating better repeatability of measurements when biopsies were sonicated before further processing, and also indicating that sonication did not interfere with SERCA activity (data not shown). Hence, sonication was performed in all further optimization measurements. The interday CVs of the optimized SERCA activity assay of two different samples were 5.0% (68.8 ± 3.5 mU/mgP) and 5.7% (93.9 ± 5.3 mU/mgP). The intraday CVs of the same two samples were 4.2% (293.3 ± 12.3 mU/ml) and 5.7% (289.3 ± 11.32 mU/ml). These CVs all indicate high reproducibility (CV < 10%) [26].

**Fig. 2:**
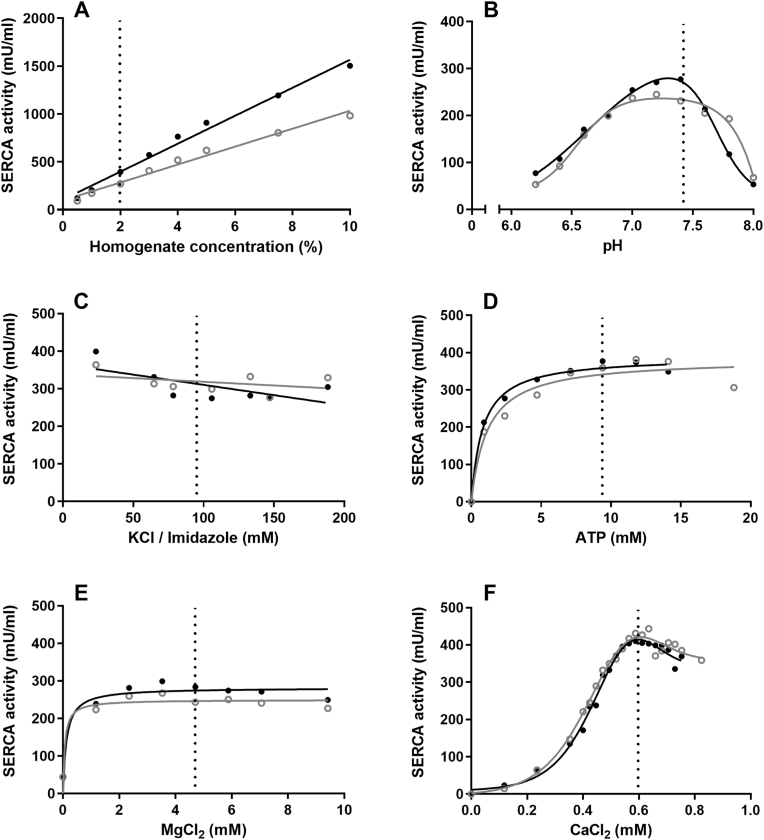
Optimization of reaction conditions of the SERCA enzyme assay. Effects of homogenate (A), pH (B), KCl/Imidazole (C), ATP (D), MgCl_2_ (E), and CaCl_2_ (F) concentrations on SERCA activity. Protein normalization was not performed for these experiments, as identical volumes of the same muscle homogenate were used for each condition; results are therefore expressed in mU/mL reaction mixture rather than mU/mg protein. Black and grey circles and regression lines represent two different samples from healthy subjects. The dotted lines represent the reaction mix concentrations chosen for further measurements, which consist of 2% homogenate, 94.1 mM KCl/imidazole, 9.4 mM ATP, 0.588 mM CaCl_2_, and 4.7 mM MgCl_2_, with pH of 7.4. These concentrations were used in all sub-experiments (A-F), except for concentrations of 4.7 mM ATP and 0.518 mM CaCl_2_ in the pH (B), KCl/Imidazole (C), and MgCl_2_ (E) curves. For the regression lines we used linear regression (A + C), Bell-shaped (B + F), and Michaelis-Menten (D + E).

**Fig. 3:**
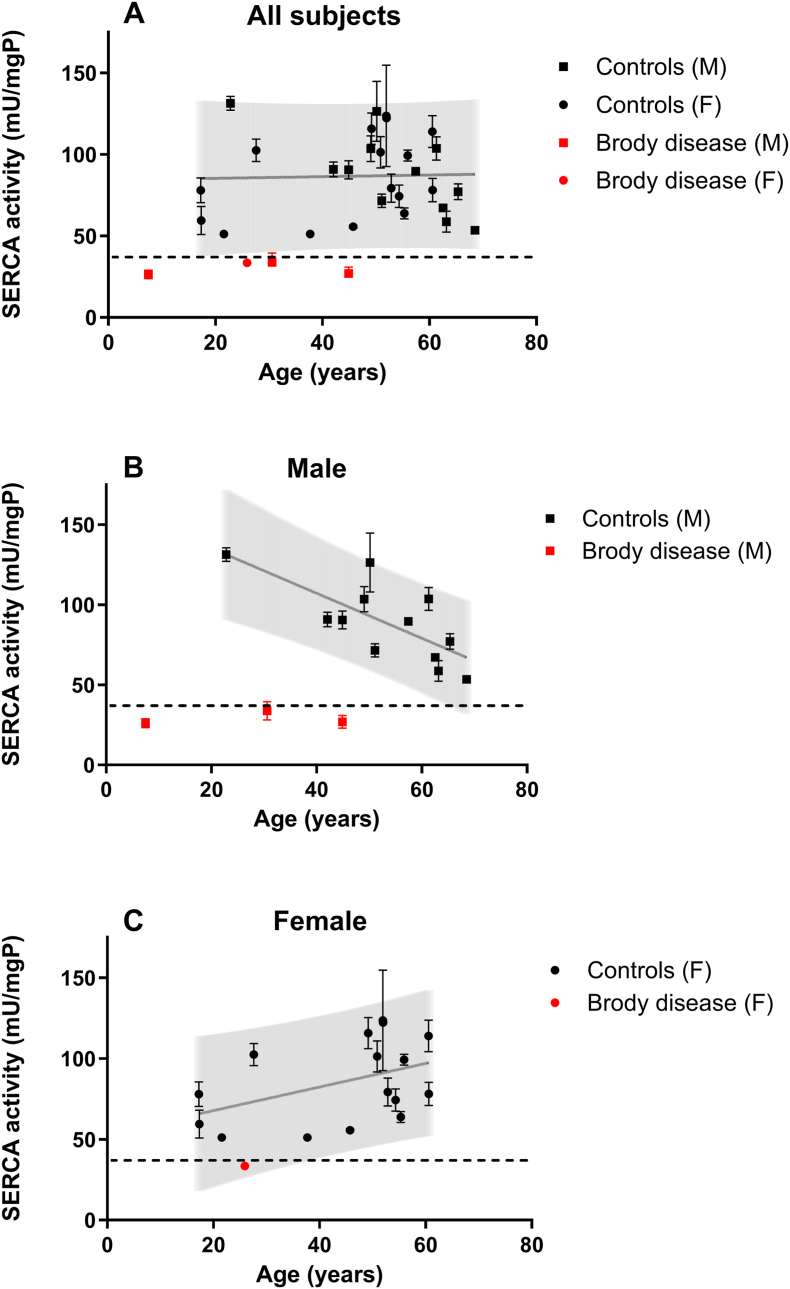
SERCA activity in healthy controls and patients with Brody disease. Results are displayed for male and female subjects together (A) and separately (B + C). The grey shaded areas represent the 90% prediction bands. The horizontal dashed lines represent the lower limit of normal at 36.6 mU/mgP (mean all controls – 2xSD). For males there is a significant effect of age on SERCA activity (P = 0.007, r^2^ = 0.529), which is not seen in females (P = 0.097, r^2^ = 0.185). For both sexes together there is no age effect (P = 0.880, r^2^ = 0.001). Error bars represent the Standard Error of the Mean (SEM) of the duplicate measurements for each individual subject. When no error bars are shown, it indicates that there was insufficient material to perform duplicate measurements for that given subject. mgP, milligrams of protein; SERCA, sarcoplasmic/endoplasmic reticulum ATPase.

The effects of pH, homogenate concentration, and different buffer compositions on SERCA activity are displayed in Fig. 2. For each of these factors an optimum concentration was determined. For homogenate and KCl/Imidazole concentrations this was arbitrarily determined at 2% and 94.1 mM, respectively (Fig. 2A and C), in line with the previous assay [8]. For all other buffer constituents, the concentration producing the highest SERCA activity was chosen. This resulted in an optimized SERCA activity assay with 2% muscle homogenate and buffer concentrations of 94.1 mM KCl/imidazole, 9.4 mM ATP, 0.588 mM CaCl_2_, and 4.7 mM MgCl_2_ with pH of 7.4 (Fig. 2A–F). These concentrations were used for the measurements of the samples from healthy controls and patients with Brody disease (Fig. 3).

### 3.2 SERCA activity in healthy controls and Brody disease

The measured SERCA activity in muscle samples from healthy subjects and Brody disease patients is displayed in Table 1 and Fig. 3. SERCA activity in male (Fig. 3B), female (Fig. 3C) and all control subjects combined (Fig. 3A) was normally distributed (*P* = 0.689, *P* = 0.145, and *P* = 0.140, respectively). Control male and female subjects had similar SERCA activity (Table 1, 88.6 ± 24.7 vs 85.6 ± 25.7 mU/mgP, respectively; *P* = 0.756). For both sexes combined, the average SERCA activity was 86.7 ± 25.1 mU/mgP, resulting in an overall lower limit of normal of 36.6 mU/mgP (mean – 2xSD). There was a significant decreasing effect of age on SERCA activity in male controls which was not found in female subjects (Fig. 3B and C). All patients with Brody disease showed a decreased SERCA activity (Table 1 and Fig. 3A–C).

The optimized SERCA assay achieved a high level of diagnostic accuracy to differentiate healthy controls from patients with Brody disease with an area under the receiver operating characteristic (ROC) curve of 1.0 (95% CI: 1.0-1.0; *P* = 0.001).

## 4 Discussion

This study has extensively evaluated and optimized all parameters of the SERCA activity assay in human whole muscle homogenates. Based on an assay developed in 1992 [9], we have optimized the sample preparation and thoroughly evaluated the assay conditions. With this robust enzyme assay we were able to provide reference values which were validated by confirming markedly low SERCA activity in Brody disease muscle samples.

### 4.1 Optimizing SERCA activity assay

The first step to improve the reproducibility of the assay compared to previously described methods, i.e., as described in Benders et al. [8,9], was the sonication of muscle homogenates. This markedly lowered the within-sample variability compared to manual homogenization with a potter-Elvehjem homogenizer. Other important changes to the SERCA activity assay were the optimization of the ATP and calcium concentrations. This resulted in a more robust assay that is relatively tolerant to minimal variations in calcium and ATP concentrations. Overall, the measured SERCA activity was lower than previously published, e.g. Refs. [27,28]. However, our assay is not designed to provide the absolute maximal value of SERCA activity but to detect differences in activity between Brody disease patients and healthy controls. This assay is optimized for a clinical diagnostic settings, where simplicity, ease of implementation, and high-throughput capability are essential, focusing on diagnostic differentiation rather than assessing the maximal enzymatic activity of SERCA.

### 4.2 SERCA activity in healthy controls and Brody disease

Our method of SERCA activity assessment was validated by the measurement of muscle samples from patients suffering from Brody disease. The SERCA activity in all Brody disease samples was markedly decreased in comparison to the reference range obtained from measurements in muscle samples from healthy controls, demonstrating the discriminative ability of the assay (Fig. 3).

### 4.3 Clinical implications

Before the introduction of next generation sequencing (NGS; techniques such as whole exome and gene panel sequencing) measurement of SERCA activity was mainly used to guide clinicians and geneticists in the diagnostic workup, i.e., decreased SERCA activity followed by Sanger sequencing of the *ATP2A1* gene. Nowadays a muscle biopsy is often initially omitted, and instead NGS is performed as a first step when a patient is suspected of suffering from a myopathy [[29], [30], [31]]. However, the muscle biopsy still plays an important role as follow-up test to evaluate the pathogenicity of genetic variants of unknown clinical significance. For *ATP2A1* variants of uncertain significance, decreased SERCA activity can provide supportive evidence for the diagnosis of Brody disease, particularly in cases with atypical or variant presentations of the Brody phenotype, as described in recent case reports [32,33]. Furthermore, specific morphological and biochemical changes can guide genetic testing. This is especially relevant in hereditary myopathies that are difficult to detect using regular exome/genome sequencing, e.g., mitochondrial DNA mutations, repeat expansions, and deletions [[34], [35], [36]]. Decreased or low-normal SERCA activity should prompt the geneticist to perform an in-depth search for mutations in the *ATP2A1* gene and/or SERCA1 Western blot analysis. This was demonstrated in the female patient with Brody disease of this study in which initially only a single heterozygous *ATP2A1* variant was identified using NGS. However, after the SERCA activity was found to be markedly decreased, extensive genetic analysis using *ATP2A1* Sanger sequencing revealed an additional pathogenic large-scale deletion, confirming the diagnosis of Brody disease [4].

Western blot analysis of the SERCA1 protein can have an added value to SERCA activity measurements in the diagnostic workup of Brody disease [4]. Both techniques are complementary: western blots provide information on SERCA1 protein content but not on its function and vice versa for the SERCA activity assay. In our recent paper describing a large cohort of patients with Brody disease [4], we found decreased SERCA1 content in all assessed muscle samples using Western blot, indicating that the presence of the mutation(s) probably destabilize the transcript/protein. However, it is possible that certain *ATP2A1* mutations, e.g., missense variants in the catalytic domain of the SERCA protein, only affect SERCA1 function but not protein content, resulting in a normal Western blot and abnormal SERCA activity profile. Measurement of a larger number of Brody disease muscle samples with both SERCA activity and Western blot is needed to further test this hypothesis.

### 4.4 Age-related decreased SERCA activity

We observed that SERCA activity decreased with age in healthy male controls but not in women, which is in agreement with previously published observations [8]. It is important to note that the average age in the male subjects was higher compared to the female subjects. Therefore, we cannot exclude a possible decrease of SERCA activity in women above the age of 60 years old.

A similar male-specific age effect was described for in vivo muscle relaxation rate in human elbow flexors [37]. These findings could be explained by age-related atrophy of type II (fast twitch) muscle fibers in men which has been demonstrated in quadriceps, e.g., Refs. [[38], [39], [40], [41], [42]], and other muscle groups, e.g., Refs. [43,44]. In contrast, in women there is scarce information on age-related morphological changes in quadriceps muscle. We identified one study that showed decreased type II fiber area in elderly compared to young women with an age-related decrease of 0.6% and 13.1% of the type II cross sectional area (CSA) for peripheral and central muscle fibers, respectively [45]. However, these changes appear not as marked as the type II atrophy seen in men, e.g., 35% smaller CSA [41]. On the basis of these data, we hypothesize that the observed sex-specific age effect on compound SERCA activity is due to age-related atrophy of type II muscle fibers which is more pronounced in males than females.

### 4.5 Limitations

First, with the measurement of compound SERCA activity there is no differentiation between SERCA1 and SERCA2 activity. Therefore, a decreased result could be caused by lowered SERCA1 and/or SERCA2 activity. However, so far, no myopathy has been associated with SERCA2 dysfunction [5,6]. Furthermore, in quadriceps muscle the proportion of SERCA2 is significantly lower than SERCA1 resulting in a limited effect of any potential decreased SERCA2 activity [8,10].

Second, our assay is not fully specific for SERCA since the Plasma Membrane Ca^2+^ ATPase (PMCA) is expected to also produce phosphate under these conditions. The use of SERCA inhibitors like thapsigargin could have enhanced the specificity of the assay [46]. However, we expect this to have had a limited effect on our measurements since PMCA only amounts to 10% of the total Ca^2+^-dependent ATPase activity and has a lower affinity for Ca^2+^ compared to SERCA [8]. Furthermore, we found that the current assay provided satisfactory differentiation between Brody disease patients and controls without the need for specific SERCA inhibition, keeping the assay as simple as possible for clinical diagnostic purposes.

Third, muscle samples from two patients with Brody disease were obtained from deltoid muscle instead of quadriceps muscle. Thus, we cannot exclude that the decreased SERCA activity in these samples is partly due to deltoid specific decreased SERCA activity. However, we deem this unlikely with deltoid muscles having a similar proportion of type II fibers as quadriceps muscles (43 and 41%, respectively) [47,48]. Normative data on SERCA activity across different human muscle groups warrants further investigation.

Fourth, we did not gather normative data on SERCA activity above the age of 70 years old which would be insightful in the study of age-related physiological changes in muscle functioning. This is, however, not likely a problem in the diagnosis of Brody disease because of the average young age of symptom onset [4].

Fifth, the number of Brody disease patients included in this study was limited due to the extreme rarity of this myopathy. However, given that our cohort represents approximately 10% of all reported cases worldwide, we believe that the findings provide valuable and meaningful insights into this rare condition [4].

Lastly, our results show high discriminate power to differentiate patients with Brody disease from healthy subjects. However, it is possible that SERCA activity is also abnormal in other (calcium related) myopathies, such as *RYR1*-related myopathy and tubular aggregate myopathy linked to *STIM1*, *ORAI1*, and *CASQ1* mutations [[49], [50], [51], [52], [53]]. Additionally, previous research demonstrated an increase in SERCA activity after 12 weeks of high-resistance training [54]. Therefore, it is possible that profound muscular disuse could also decrease SERCA activity. Future research on SERCA activity in these subject groups is needed to determine the specificity of abnormal SERCA activity.

## 5 Conclusion

In conclusion, we have developed a robust enzyme assay to measure SERCA activity with high discriminative power to distinguish patients with Brody disease from healthy controls. The measurement of SERCA activity can further increase our understanding of calcium handling in healthy muscle, Brody disease, and other (calcium related) myopathies.

## Funding

This research received no external funding.

## CRediT authorship contribution statement

**J.P. Molenaar:** Conceptualization, Data curation, Formal analysis, Investigation, Methodology, Validation, Writing – original draft, Writing – review & editing. **M.M. Snoeck:** Methodology, Resources, Writing – review & editing. **S. Treves:** Conceptualization, Data curation, Writing – review & editing. **B.J. Stoltenborg:** Investigation, Methodology, Resources, Validation, Writing – review & editing. **K.C. Wethly:** Investigation, Methodology, Resources, Validation, Writing – review & editing. **A. Oosterhof:** Investigation, Methodology, Writing – review & editing. **E.J. Kamsteeg:** Investigation, Methodology, Supervision, Writing – review & editing. **D. Sternberg:** Data curation, Investigation, Methodology, Writing – review & editing. **J. Doorduin:** Conceptualization, Writing – review & editing. **B.G. van Engelen:** Conceptualization, Data curation, Methodology, Writing – review & editing. **N.C. Voermans:** Conceptualization, Data curation, Formal analysis, Investigation, Methodology, Resources, Supervision, Validation, Writing – review & editing. **R.J. Rodenburg:** Conceptualization, Data curation, Formal analysis, Methodology, Writing – original draft, Writing – review & editing.

## Declaration of competing interest

The authors declare that they have no known competing financial interests or personal relationships that could have appeared to influence the work reported in this paper.

## Data Availability

Data will be made available on request.
